# Neural network-crow search model for the prediction of functional properties of nano TiO_2_ coated cotton composites

**DOI:** 10.1038/s41598-021-93108-9

**Published:** 2021-07-01

**Authors:** Nesrine Amor, Muhammad Tayyab Noman, Michal Petru, Aamir Mahmood, Adla Ismail

**Affiliations:** 1 Department of Machinery Construction, Institute for Nanomaterials, Advanced Technologies and Innovation (CXI), Technical University of Liberec, Studentská 1402/2, 461 17 Liberec 1, Czech Republic; 2 Department of Material Engineering, Faculty of Textile Engineering, Technical University of Liberec, Studentská 1402/2, 461 17 Liberec 1, Czech Republic; 3 Electrical Engineering Department, Laboratory of Signal Image and Energy Mastery (SIME, LR 13ES03), University of Tunis, ENSIT, 1008 Tunis, Tunisia

**Keywords:** Computational science, Computer science, Materials for devices, Materials for energy and catalysis, Nanoscale materials

## Abstract

This paper presents a new hybrid approach for the prediction of functional properties i.e., self-cleaning efficiency, antimicrobial efficiency and ultraviolet protection factor (UPF), of titanium dioxide nanoparticles (TiO_2_ NPs) coated cotton fabric. The proposed approach is based on feedforward artificial neural network (ANN) model called *a multilayer perceptron (MLP)*, trained by an optimized algorithm known as crow search algorithm (CSA). ANN is an effective and widely used approach for the prediction of extremely complex problems. Various studies have been proposed to improve the weight training of ANN using metaheuristic algorithms. CSA is a latest and an effective metaheuristic method relies on the intelligent behavior of crows. CSA has been never proposed to improve the weight training of ANN. Therefore, CSA is adopted to optimize the initial weights and thresholds of the ANN model, in order to improve the training accuracy and prediction performance of functional properties of TiO_2_ NPs coated cotton composites. Furthermore, our proposed algorithm i.e., multilayer perceptron with crow search algorithm (MLP-CSA) was applied to map out the complex input–output conditions to predict the optimal results. The amount of chemicals and reaction time were selected as input variables and the amount of titanium dioxide coated on cotton, self-cleaning efficiency, antimicrobial efficiency and UPF were evaluated as output results. A sensitivity analysis was carried out to assess the performance of CSA in prediction process. MLP-CSA provided excellent result that were statistically significant and highly accurate as compared to standard MLP model and other metaheuristic algorithms used in the training of ANN reported in the literature.

## Introduction

Titanium dioxide (TiO_2_) in different nano dimensions i.e., nanoparticles^1^, nanorods^2^, nanobelts^3^, nanowires^4^, nanosheets^5^ and nanoflowers^6^ have shown prestigious trend as a photocatalyst and a multifunctional coating material in many fields of life especially in textiles. Nano TiO_2_ is significantly utilised in photocatalytic self-cleaning^7^, antimicrobial coatings^8^, superhydrophilic surfaces^9^ and waste water treatment^10^. TiO_2_ is an intrinsic n type metal oxide semiconductor material that closely resembles with zinc oxide in photocatalytic properties. The prominent features that enables TiO_2_ as a functional material in many applications are photocatalytic activity, chemical stability and non-toxicity^11^. Researchers have synthesized and coated nano TiO_2_ on textile substrates in order to achieve various properties based on photocatalytic activity^12^. In an experimental study, Noman et al. successfully synthesized and coated TiO_2_ nanoparticles on cotton fabric under sonication method. The developed composites were evaluated for self cleaning and antimicrobial characteristics against methylene blue (MB) dye and bacteria culture i.e., Staphylococcus aureus and Escherichia coli respectively. The developed composites showed excellent results for self-cleaning and antimicrobial properties. The experimental design and the obtained results were tested under regression model for statistical evaluation through Design Expert (DE) software^13^. Here, in this current study, an attempt has been made to develop a prediction model by using machine learning tools that can work in two ways i.e., correlates the actual response of coated fabric with process variables, analyse the predicted response of DE and indicate which approach is better as a prediction model in reality. Nowadays, artificial neural network (ANN) exhibits a strong advantage in capturing any type of existing relationship from given data as it does not include a physical mechanism and a mathematical model^14^. Thanks to the training process, ANN can learn, understand and recognize the information treatment rules, adapt and predict the wanted output variables from database considered as input variables^15,16^.

In general, textile processes are mostly non-linear in nature and a lot of efforts are applied to obtain optimal solutions^17–20^. ANN is an excellent approach that has been widely used for the prediction of various properties of textile materials where it has proven its effectiveness and potential, such as: prediction of the tensile properties of even and uneven yarns extracted from polyester-cotton blend^21^; prediction of the warp and weft yarns crimp in woven barrier fabrics^22^; prediction of antimicrobial performance of chitosan/AgCl-TiO_2_ coated fabrics^23^; prediction of core spun yarn strength, elongation and rupture^24^; prediction of cotton fibre^25^; prediction the change of shade of dyed knitted fabrics^26^; prediction of coatings process on textile fabrics^27^; and prediction of thermal resistance of wet knitted fabrics^28^. These mentioned work reveal that the most common type of ANN algorithm used in textile industry is multilayer perceptron MLP^29,30^. MLP is a class of feedforward ANN that has the advantages of self-learning, high nonlinearity resolution and the ability of mapping between input and output variables without introducing a mathematical model between nonlinear data and precisely predict the best function. However, the main drawbacks of MLP model are slow convergence rate, hard understanding problem and stuck in the local minimum values.

The use of metaheuristic algorithms has gained attention of scientists and researchers in order to improve the performance of ANN during training process. Gao et al. proposed a dendritic neuron model (DNM) by taking into account the nonlinearity of synapses^31^. They used six algorithms to train the DNM that include genetic algorithm, biogeography-based optimization, particle swarm optimization, ant colony optimization, population-based incremental learning and evolutionary strategy. Here, simulations results showed that DNM trained by biogeography-based optimization is the most effective for enhancing DNM performances. Wang et al. proposed a modified version of the gravitational search algorithm (GSA) based on the hierarchy and distributed framework^32^. Then, they tested GSA to train the MLP, where they showed promising results. Xiao et al. used a hybrid approach that combined ANN and genetic algorithm (GA) in order to predict cotton-polyester fabric pilling^33^. The proposed hybrid approach showed good results compared to the standard ANN. Hussain et al. compared ANN with adaptive neuro-fuzzy inference system (ANFIS) in the evaluation of fabrics wrinkle recovery^34^. The simulation results demonstrated that ANN performed a slightly better than ANFIS with significant accuracy percentage. However, ANFIS process was more useful while drawing surface plots among input and output variables. Dashti et al. predicted the yarn tenacity using ANN trained by genetic algorithm. The performance of this approach was useful to achieve desired tenacity with minimum production cost. However, it is a time-consuming process^35^. Abhijit et al. applied a combination of GA and ANN as a hybrid algorithm to predict comfort performance and the range of ultraviolet protection factor (UPF)^36^. ANN was applied as a prediction tool and GA was utilized as an optimization tool and for experimental purpose, a set of four samples were selected for the evaluation of functional properties. The proposed ANN–GA method was carried out for an iterative set of variables until the required fabric properties achieved. Ni et al. proposed a novel online algorithm that detects and predicts the coating thickness on textiles by hyperspectral images^37^. The proposed algorithm was based on two different optimization modules i.e., the first module is called extreme learning machine (ELM) classifier whereas, the later is called a group of stacked autoencoders.The ELM module optimized by a new optimizer known as grey wolf optimizer (GWO), to determine the number of neurons and weights to get more accuracy while detection and classification. The results explained that online detection performance significantly improved with a combination of VW-SAET with GWO-ELM that provide 95.58% efficiency. Lazzús et al. used the combined ANN with particle swarm optimization (PSO) to predict the thermal properties^38^. The results demonstrated that the proposed model ANN-PSO provided better results than feedforward ANN.

Recently, a new meta-heuristic method based on the behaviors of crows has been emerged to solve complex optimization problems, known as Crow Search Algorithm (CSA)^39^. CSA include less setting parameters than the other algorithms such as GA and PSO, which make it more efficient in solving complex problems compared to other state-of-art methods^39^. The simple structure, easy implementation, and faster convergence motivate the use of CSA in the improvement of training process of ANN. Therefore, the main contributions of this paper are: (1) Proposing the use of CSA to train the MLP; (2) Investigating the accuracy of the propose MLP-CSA for the prediction of various properties of nano TiO_2_ coated cotton. The amount of titanium precursor, amount of solvent and process time were selected as input variables whereas the amount of nano TiO_2_ coated on cotton fabric, and some related functional properties i.e., self-cleaning efficiency, antimicrobial efficiency and UPF were considered as outputs variables. The achieved results were compared with the classical MLP, PSO and GA using a sensitivity analysis. Consequently, significant technical merits are satisfied which prove the effectiveness of the proposed MLP-CSA approach, thus providing an alternative solution for prediction in textiles materials applications.

The paper is organized as follows: “Material and methods” section describes the details of the materials and experimental design, as well as reviews the classical artificial neural network framework and introduces the optimized ANN model with crow search algorithm. In “Results and discussion” section, simulation, comparison and discussion results of prediction of functional properties of nano TiO_2_ coated cotton fabric are presented. Finally, “Conclusions” section summarizes the main findings and concludes the paper.

## Material and methods

### Material and experimental

Bleached cotton fabric with GSM (fabric mass) 110 g m^−2^ was used. Titanium tetrachloride, isopropanol and MB dye were taken from sigma aldrich. The selected variables were the amount of titanium precursor (titanium tetrachloride); the amount of solvent (isopropanol) and sonication time. The combination of variables is illustrated in Table 1.

**Table 1:** The input variables and experimental design.

Sample	TiCl_4_ (ml)	C_3_H_7_OH (ml)	Sonication time (h)
1	10	6	0.5
2	6	2	3
3	10	6	2
4	6	4	3
5	6	4	2
6	6	4	3
7	10	2	4
8	6	4	1
9	6	4	1
10	10	4	3
11	2	2	0.5
12	2	6	4
13	6	4	2
14	2	6	0.5
15	6	4	1
16	6	4	4
17	6	6	2
18	2	4	1
19	2	2	4
20	10	2	0.5

### Artificial neural network

ANN are mathematical models inspired from biological nerve systems that are responsible for the functionality of human brain. A very special feature of ANN is the automatically creation, derivation and exploration of new information using previous learning that is called as training process^40^.

Multilayer perceptron (MLP) is one of the most common and typical learning algorithm in ANN that deals with non-linear models by reducing the wanted target error in a gradient descent pattern though tailoring the weight factors and biases^33,41^. In this algorithm, training occurs in three steps: 1) Forward propagation step: an experimental data is introduced to ANN as input and its effect is propagated in different stages through different layers of the network. Then, as a result, the outputs are generated. 2) Computation of the error: the error vector is computed from the difference between predicted and actual outputs. 3) Backward propagation step: The computed error vector is propagated backwards to the ANN and the synaptic weights are adjusted in such a way that the error vector reduces with every iterative step. Furthermore, the ANN model is getting closer and closer to generating the desired output.

**Figure 1:**
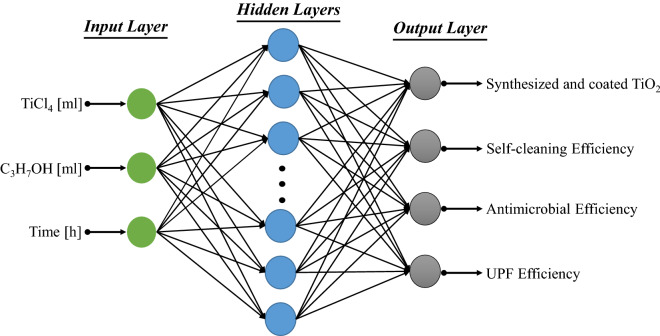
MLP model for the prediction of functional properties of nano TiO_2_ coated cotton.

Technically, ANN are used to model non-linear problems in order to predict output dependent variables $y= [y_{1}, \ldots , y_{n}]$ for given independent input variables $x= [x_{1}, \ldots , x_{k}]$ from their training values. The obtained results mainly depend on weights $w= [w_{1}, \ldots , w_{k}]$. The following equation represents the relationship between input and output of the network^42,43^:

1 $$ y =\varphi \left( \sum _{j} w_j *x_j +b \right) $$

where, *y* is the output. $x_j$ is the *j*th input. $w_j$ is the *j*th weight and *b* represents the bias. $\varphi$ is the activation function. The biases and weights comprise the information that the neuron recovers during the training phase. A detail theoretical discussion of ANN architecture and training algorithms are presented by different researchers in their studies^15,44,45^. Theoretically, by increasing number of network layers, ANN generates significantly accurate results. However, increasing number of network layers is a time consuming process and makes the training process difficult to fit. Therefore, we adopted the standard structure of feedforward ANN, i.e., MLP model that includes three-layers, one input layer; one hidden layer, and one output layer for the prediction of functional properties (see Fig. 1).

There were training and testing parts in the proposed MLP model and 75% of the data from Table 1 was used for the training of the proposed model whereas 25% of the data was used for testing purpose respectively. Three physical factors shown in Table 1 were considered as training inputs vectors. Therefore, the number of input nodes for training was 3 and the number of nodes for output layer was 4. The random selection of a number of hidden neurons might cause either overfitting or underfitting problems. To avoid these problems, the number of nodes for hidden layer was calculated according to the following equation^33^:

2 $$ N=\sqrt{m+n}+a $$

where *N* is the number of hidden layer nodes. *m* and *n* represent the number of input and output nodes, respectively. *a* is a constant with a value range [1, 10].

### Optimized ANN model with crow search algorithm

- **Crow search algorithm**

Crow search algorithm (CSA) is a recent meta-heuristic method proposed in^39^ to solve constrained engineering problems. The structure of this algorithm is modeled in mathematical expressions inspired from the intelligent behavior of the crows while saving their excess food in hiding places and retrieves it when the food is needed. The basic principle of crows is to observe food spots for other birds, and steal them when the birds leave their place. Furthermore, if the crow commits the theft, it will take additional precautions such as moving to new hiding places to avoid becoming a victim in the future again.

The position of crows *x* is given by:

3 $$ x^{i,k}=[x_{1}^{i,k}, x_{2}^{i,k},\ldots ,x_{d}^{i,k}]. $$

where *i* = 1, 2, ..., *N* is the indices of crow *i* and *N* is the number of crows. $k =1,2,\ldots , k_{max}$ is the indices of iterations, $k_{max}$ is the maximum number of iterations and *d* is a dimensional environment.

The crows move through their habitats and seek better hiding places to keep their food safe from thieves. Every crow has an intelligent memory that allows it to memorize all its previous food hiding places. One of the important activities of the crow *i* is following crow *j* by getting close to where the food is hiding and stealing it. Therefore, two main events may occur in CSA:

- Case 1: Crow *j* has no idea that crow *i* is following it. Here, crow *i* will get close to the hiding place and changes its position to a new position as follows:
  4 $$ x^{i,k+1}= x^{i,k}+r_{i} . fl^{i,k} . (m^{j,k}-x^{i,k}) $$
  where $r_{i}$ represents a random value range [1, 10]. $fl^{i,k}$ represents the flight length of crow *i* at iteration *k*. $m^{j,k}$ is the best position visited by crow *j* at iteration *k*.
- Case 2: Crow *j* discovers that crow *i* is followed it. Here, to protect its hideout from theft, crow *j* will change the flight pass to mislead followers by moving to another position in its environment.

Therefore, the position of each crow can be described using the following expression:

5 $$x^{i,k+1}= \left\{ \begin{array}{ll} x^{i,k}+r_{i} . fl^{i,k} . (m^{j,k}-x^{i,k})&{\quad} r_j \ge AP^{j,k}\\ {a\,random\,position} &{\quad} {otherwise} \end{array} \right. $$

where $AP^{j,k}$ represents the awareness probability of crow *i* at iteration i.

- **Optimized ANN model with crow search algorithm**

The MLP has many drawbacks such as slow convergence rate and stuck into the local minima. The training process and the convergence rate of MLP become more accurate when it is trained using a metaheuristic approaches like PSO^38,46–48^ and GA^33,49^. However, CSA has been never investigated in training ANN. Training process comprises determining the collection of corresponding influences that depreciate the training error. Therefore, we proposed a new model based on combination between the CSA approach and MLP model to predict functional properties of nano TiO_2_ coated cotton. The main objective is to find the optimal prediction of the wanted outputs while minimizing the error results in training process of MLP. In this proposed model, MLP model optimized by CSA where CSA is used to optimize the weight and threshold values of MLP, which can more accurately predict the output results.

In this context, at every iteration *k*, the crow position $x^{i,k+1}$ is considered as the collection of weights in MLP-CSA. The mean square error (MSE) between the predicted and actual outputs is considered as fitness value for the proposed algorithm MLP-CSA, and the CSA attempts to minimize it during the training of MLP:

6 $$ Minimize \{F\} = min \{\frac{1}{N}\Sigma _{i=1}^{N}{\Sigma _{j=1}^{n}{(y_{ij}-{\hat{y}}_{ij})^{2}}}\} \; \; \; where \; \; i=1,\ldots ,N\; j=1,\ldots ,n. $$

where *N* and *n* are the number of training samples and the number of output nodes, respectively.

**Figure 2:**
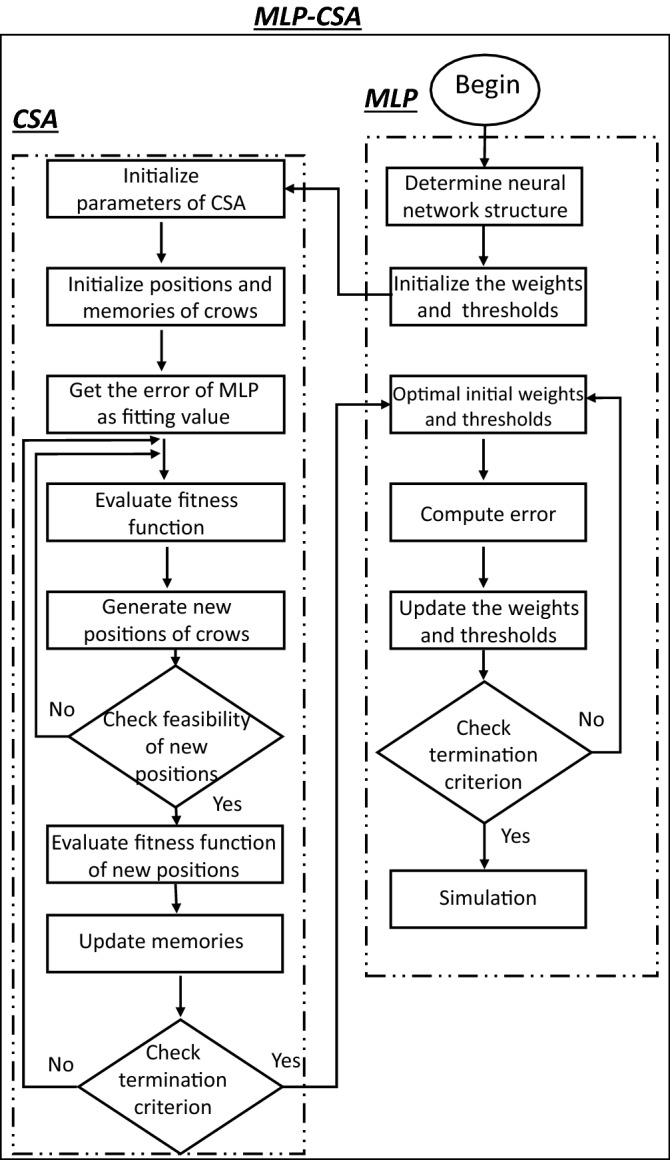
MLP-CSA model for the prediction of functional properties of nano TiO_2_ coated cotton.

In main steps in this proposed MLP-CSA: (1) Initialize the parameters of algorithm: Each crow contains all the weights and thresholds of the neural network i.e., the connection weight of the input and the hidden layers, the threshold of the hidden layer, the connection weight of the hidden and the output layers, and the threshold of the output layer. (2) Initialization of the position and memory of every crow: MLP-CSA is started with random initialization of crow positions (bias and weights). Here, every crow is moved into the weighted search space striving for propagating the error. (3) Evaluate fitness function: The initial bias and weights used in the learning process to compute the initial training error. (4) Generate new position: random selection and following another crow to find its position of the hidden food. The new positions of crows are given using Eq. (5). (5) Evaluate fitness function of the new obtained positions and update the memory of each crow: computing the fitness function value for every crow according to the new position. Then, update the memory of each crow if the evaluation of the fitness function value of each crow is better than the previous memorized fitness function value. Figure 2 presents the flowchart of the proposed algorithm MLP-CSA for the prediction of functional properties of nano TiO_2_ coated cotton.

### Robustness analysis

The performance of the proposed MLP-CSA model was evaluated using various statistical indicators i.e., root mean squared error (RMSE), mean absolute error (MAE) and coefficient of determination ($R^{2}$), defined respectively by the following equations:

7 $$ RMSE=  \sqrt{\frac{1}{n}\Sigma _{i=1}^{n}{(y_i-{\hat{y}}_i)^2}} $$

8 $$ MAE=  \frac{1}{n}\Sigma _{i=1}^{n}\left| (y_i-{\hat{y}}_i)\right| $$

9 $$ R^{2}= \left( \frac{ \sum _{i=1}^{n}(y_i-{\bar{y}})(\hat{y_i}-\bar{{\hat{y}}}) }{ \sqrt{\sum _{i=1}^{n}(y_i-{\bar{y}})^2}\sqrt{\sum _{i=1}^{n}(\hat{y_i} -\bar{{\hat{y}}})^2}}\right) ^{2} $$

where $y_i$ and ${\hat{y}}$ represent the actual and network outputs, respectively. *n* is the number of samples. ${\bar{y}}$ represents the mean of the actual variables and $\bar{{\hat{y}}}$ is the mean of the predicted variables.

### Sensitivity analysis

Sensitivity analysis (SA) is a statistical method that provides an idea of how sensitive is the best solution chosen to any changes in input values from one or more parameters^50^. ANOVA is an independent SA method that assesses if there is any statistically significant association between one or more inputs and output^51–54^. ANOVA utilizes the statistic ratio *F* to define if there is a significant difference exists between the average responses to main interactions or interactions between factors. The higher *F* value indicates higher rankings. The *p* value represents the differences between column means if they are significant or not. In this paper, one-way ANOVA is used to assess the correlation between obtained results of coated fabric with process variables using the proposed MLP-CSA, MLP-PSO, MLP-GA, standard MLP model and experimental values.

A repeated measures ANOVA test followed by a post-hoc Bonferroni multiple comparisons test (with an alpha level significance value of 0.05) is a technique used to evaluate whether there is any statistically significant difference between two or more independent groups^55^. Therefore, these tests were used to compare the differences in the prediction errors between the results obtained by all algorithms and to determine which algorithm would be different from the others.

## Results and discussion

We predicted the functional properties of nano TiO_2_ coated cotton fabric using the optimized MLP model with crow search algorithm (MLP-CSA). The results obtained with the proposed MLP-CSA were compared with the standard MLP model, optimized MLP model with genetic algorithm (MLP-GA) and optimized MLP model with particle swarm optimization (MLP-PSO).

### Parameters setting of the proposed MLP-CSA model

The selected MLP model with three-layers (i.e., an input, a hidden, and an output layers) was adjusted in a way that the number of hidden layer nodes could not exceed the range of values [4, 13] according to Eq. (2). We found that the best number of hidden layer nodes are 11, where the network provides highly accurate results. Therefore, we adopted 11 hidden layer nodes to be used in this work. The parameters and settings of the MLP training network, optimized models with CSA, PSO and GA are presented in Table 2.

**Table 2:** Parameters and settings of the MLP training network, optimized models with CSA, PSO, and GA.

Methods	Parameters	Settings
MLP	Training function	Trainlm
	Transfer function of hidden layer	Tansig$$(x) =2/(1+exp(-2*x))-1 $$
	Transfer function of output layer	Purelin$(x)=x$
	Learning rate	0.02
	Performance goal	0.00001
	Input node	3
	Hidden node	11
	Output node	4
CSA	Population size	55
	Awareness probability	0.1
	Flight length	2
	Number of iterations	300
PSO	Population size	50
	Inertia weight	1
	Cognitive factor C1	1.5
	Social factor C2	2
	Random values: r1, r2	[0,1]
	Number of iterations	300
GA	Population size	20
	Variation probability	0.5
	Crossover probability	0.4
	Selection method	Roulette method
	Mutation method	Float
	Crossover method	Float
	Number of iterations	300

### Comparison of MLP-CSA with currently used MLP-metaheuristics

**Figure 3:**
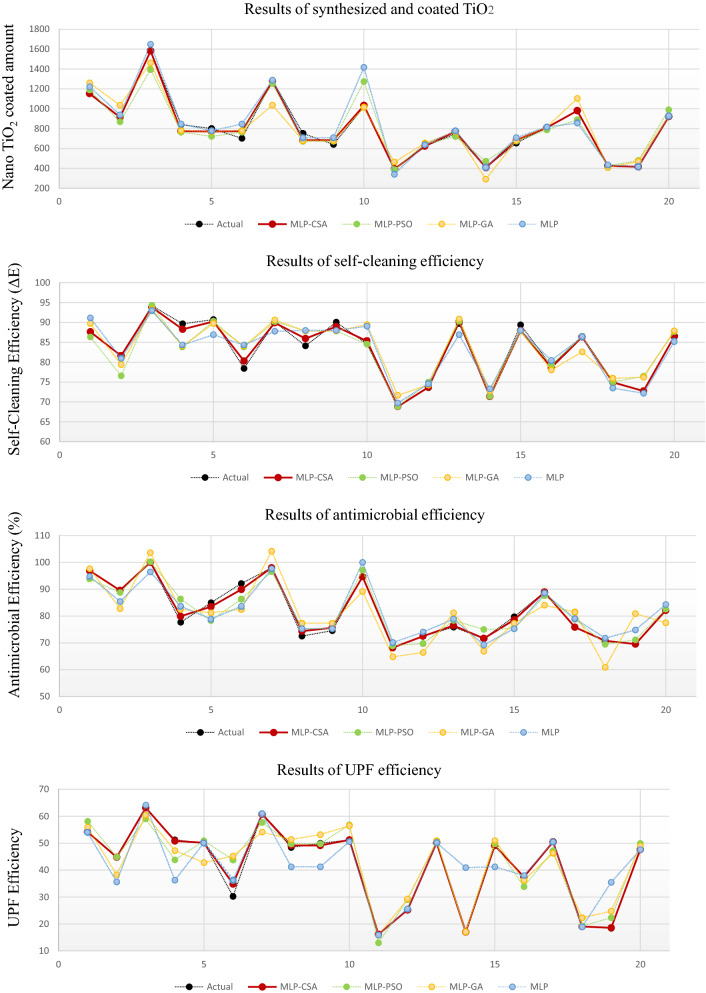
Simulation results for the prediction of synthesized and coated TiO_2_, self-cleaning efficiency, antimicrobial efficiency, and UPF efficiency using MLP-CSA, MLP-PSO, and MLP-GA.

The predicted values of functional properties of nano TiO_2_ coated cotton fabric are presented in Fig. 3. To confirm a fair comparison between all used algorithms, we performed many trials with different number of populations in each algorithm. Then, we considered the best results that include the lower MSE and high prediction performance for the functional properties of nano TiO_2_ coated cotton in each algorithm. The first sub-figure shows the predicted results of synthesized and coated TiO_2_; the second sub-figure represents the results of self-cleaning efficiency; the third sub-figure represents the results of antimicrobial efficiency whereas the last sub-figure illustrates the results of UPF efficiency.

**Figure 4:**
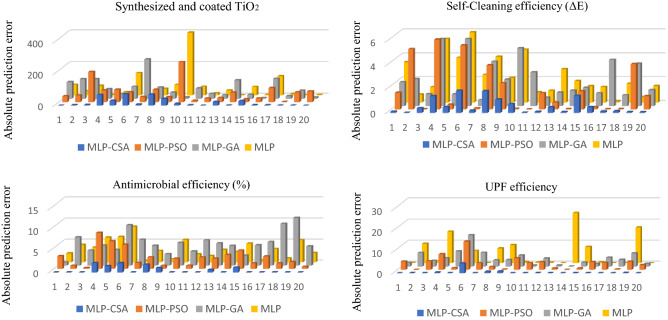
Absolute errors between predicted and actual values using MLP, MLP-CSA, MLP-PSO and MLP-GA.

The value of absolute prediction error given by the difference between predicted and actual values for all four functional properties using MLP-CSA, MLP-PSO, MLP-GA, and standard MLP are shown in Fig. 4. We observed that the values of prediction error were significantly lower for the proposed MLP-CSA as compared to MLP-PSO, MLP-GA, and standard MLP for all four functional properties.

**Figure 5:**
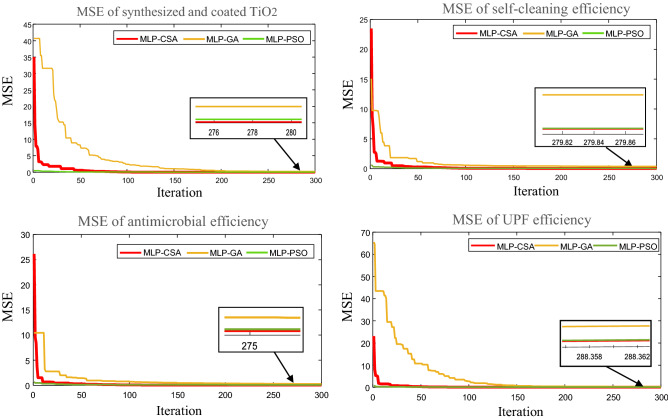
Performance MSE convergence characteristics for MLP-CSA, MLP-PSO and MLP-GA.

Figure 5 illustrates the performance MSE convergence characteristics for all used optimized models: MLP-CSA is in red, MLP-GA is in yellow, and MLP-PSO is in green. We noticed that the lower MSE values were obtained by the proposed MLP-CSA model compared to MLP-PSO and MLP-GA for all of four functional properties.

Table 3 represents the computed RMSE, MAE and R^2^ for all used models to predict the functional properties of nano TiO_2_ coated cotton fabric. We observed that the proposed MLP-CSA model provides lower errors according to RMSE and MAE, and high accuracy according to R^2^ compared to MLP-PSO, MLP-GA and standard MLP for all of the four functional properties. It is clear from the obtained results that the proposed MLP-CSA model verifies its accuracy and effectiveness in the prediction process.

**Table 3:** Errors of different approaches.

Functional properties	Methods	Training			Testing		
		RMSE	MAE	R^2^	RMSE	MAE	R^2^
Synthesized and coated TiO_2_	MLP-CSA	29.78	17.89	0.9913	30.41	18.47	0.9896
	MLP-PSO	85.92	62.68	0.9334	86.78	63.40	0.9156
	MLP-GA	89.11	67.54	91.173	89.86	68.20	0.9020
	MLP	101.47	80.49	0.8781	103.82	82.73	0.8732
Self-cleaning efficiency	MLP-CSA	0.78	0.49	0.9891	0.82	0.55	0.9876
	MLP-PSO	2.42	1.56	0.8972	2.55	1.64	0.8810
	MLP-GA	2.61	2.18	0.8683	2.80	2.30	0.8563
	MLP	2.78	2.26	0.8622	2.85	2.35	0.8517
Antimicrobial efficiency	MLP-CSA	0.86	0.49	0.9582	0.95	0.54	0.95
	MLP-PSO	3.29	2.58	0.8872	3.46	2.71	0.8853
	MLP-GA	3.66	3.11	0.8634	3.87	3.25	0.8560
	MLP	5.79	5.27	0.6703	5.90	5.34	0.6658
UPF efficiency	MLP-CSA	0.95	0.29	0.9959	1.04	0.36	0.9949
	MLP-PSO	4.20	2.95	0.9173	4.38	3.08	0.9112
	MLP-GA	4.87	3.72	0.8806	5.14	3.93	0.8775
	MLP	7.82	4.41	0.6847	8.36	4.92	0.6766

### Robustness assessment by statistical analysis

A one-way ANOVA test was applied to assess the robustness of the predicted results using MLP-CSA, MLP-PSO, MLP-GA, standard MLP as well as the experiment data. ANOVA test helps to knows how it is the correlations between the predicted responses of coated fabric with process variables. Table 4 shows the results of one-way ANOVA test of each functional properties obtained by MLP-CSA, MLP-PSO, MLP-GA, MLP and experimental. It is observed that the proposed MLP-CSA model was more statistically significant as compared to other models and experimental, as it provides the lowest *p* value for all functional properties.

**Table 4:** Analysis report of the experimental values and the predicted values using MLP, MLP-CSA, MLP-PSO and MLP-GA for functional properties of nano TiO_2_ coated cotton.

Functional properties	Methods	p value	F value
Synthesized and coated TiO_2_	MLP-CSA	2.05e−9	69.92
	MLP-PSO	1.77e−8	52.06
	MLP-GA	4.15e−7	33.26
	MLP	8.38e−7	29.99
	Experimental	8.04e−6	21.20
Self-cleaning efficiency	MLP-CSA	4.76e−6	15.96
	MLP-PSO	0.000074	14.67
	MLP-GA	0.0004	10.38
	MLP	0.0011	8.79
	Experimental	0.001	8.92
Antimicrobial efficiency	MLP-CSA	7.70e−8	42.39
	MLP-PSO	2.85e−6	24.93
	MLP-GA	7.74e−6	21.33
	MLP	6.82e−6	21.76
	Experimental	2.65e−5	17.47
UPF efficiency	MLP-CSA	3.06e−6	24.65
	MLP-PSO	7.07e−6	21.64
	MLP-GA	2.33e−5	17.86
	MLP	0.000084	14.35
	Experimental	0.00001	19.86

In addition, we performed a repeated measure ANOVA test followed by a post hoc Bonferroni multiple comparison test for all predicted error results of synthesized and coated TiO_2_, self-cleaning efficiency, antimicrobial efficiency, and UPF efficiency as shown in Tables 5, 6, 7 and 8, respectively. These tests were used to compare the differences in the prediction errors between the results obtained by all models as well as to determine which model would be different from the others. Paired sample t-tests with Bonferroni correction shows that the MLP-CSA was statistically significant and comparatively different from MLP and MLP-GA. We also noted that the MLP-CSA and MLP-PSO were statistically similar (P = 1). However, we observed that there is a minor difference in the mean error between MLP-CSA and MLP-PSO for the four functional properties as shown in Tables 5, 6, 7 and 8, which confirms that MLP-CSA provides the best results for the prediction of functional properties of nano TiO_2_ coated cotton fabric.

**Table 5:** Pairwise comparisons of all algorithms prediction error for the synthesized and coated TiO_2_.

Algorithms	Mean difference	Std. error	Sig.^b^	Lower bound	Upper bound
MLP-CSA & MLP-PSO	4.795	13.828	1.000	− 24.038	37.381
MLP-CSA & MLP-GA	8.671	15.509	.152	− 50.833	61.243
MLP-CSA & MLP	44.929*	19.035	.036	2.215	87.643

*The mean difference is significant at the .05 level.

^b^Adjustment for multiple comparisons: Bonferroni.

**Table 6:** Pairwise comparisons of all algorithms prediction error for the self-cleaning efficiency.

Algorithms	Mean difference	Std. error	Sig.^b^	Lower bound	Upper bound
MLP-CSA & MLP-PSO	− .650	.362	.992	− 1.377	.676
MLP-CSA & MLP-GA	− .706	.391	.398	− 1.773	.361
MLP-CSA & MLP	1.091	.451	.070	− .061	2.243

*The mean difference is significant at the .05 level.

^b^Adjustment for multiple comparisons: Bonferroni.

**Table 7:** Pairwise comparisons of all algorithms prediction error for the antimicrobial efficiency.

Algorithms	Mean difference	Std. error	Sig.^b^	Lower bound	Upper bound
MLP-CSA & MLP-PSO	− .532	.375	1.000	− 1.732	.667
MLP-CSA & MLP-GA	2.179*	.407	.018	1.076	3.281
MLP-CSA & MLP	− 2.628*	.773	.000	− 4.903	− .353

*The mean difference is significant at the .05 level.

^b^Adjustment for multiple comparisons: Bonferroni.

**Table 8:** Pairwise comparisons of all algorithms prediction error for the UPF efficiency.

Algorithms	Mean difference	Std. error	Sig.^b^	Lower bound	Upper bound
MLP-CSA & MLP-PSO	− .846	.568	1.000	− 2.679	.987
MLP-CSA & MLP-GA	2.728*	.823	0.036	1.056	4.400
MLP-CSA & MLP	− 1.831	1.729	.001	− 6.922	3.260

*The mean difference is significant at the .05 level.

^b^Adjustment for multiple comparisons: Bonferroni.

## Conclusions

In this paper, we proposed a novel approach based on combination between artificial neural network and crow search algorithm (MLP-CSA), where CSA has been used for the first time to improve the training process of MLP. The proposed approach has been applied to predict the functional properties of nano TiO_2_ coated cotton. The obtained results showed a higher prediction accuracy for the proposed MLP-CSA model compared to standard MLP, MLP-PSO and MLP-GA. The computed values of RMSE and MAE from all predicted results confirm that the proposed MLP-CSA model has lower error and more statistically significant as compared to all three other models. The successful utilization of the developed model reveals a non-linear relationship between the selected parameters for the prediction of functional properties. The findings of this work highlight that MLP-CSA approach can be effectively used for the prediction of other properties of nano coated fabrics.
